# The Effectiveness of Rehabilitation after Open Surgical Release for Trigger Finger: A Prospective, Randomized, Controlled Study

**DOI:** 10.3390/jcm12227187

**Published:** 2023-11-20

**Authors:** Taichi Saito, Ryo Nakamichi, Ryuichi Nakahara, Keiichiro Nishida, Toshifumi Ozaki

**Affiliations:** Department of Orthopaedic Surgery, Okayama University Graduate School of Medicine, Dentistry and Pharmaceutical Sciences, 2-5-1, Shikata-cho, Kitaku, Okayama 700-8558, Japan

**Keywords:** hand surgery, rehabilitation, open surgical release, trigger finger

## Abstract

Background: It is not clear whether rehabilitation after surgery for trigger finger is effective. The aim of this study was to reveal its effectiveness for trigger finger. Methods: This study was a randomized, controlled trial that included patients who underwent operations for trigger fingers. The patients in the rehabilitation group had postoperative occupational therapy (OT) for 3 months, while the patients in the control group were not referred for rehabilitation but received advice for a range of motion exercises. We evaluated the severity of trigger finger, Disability of Arm-Shoulder-Hand (DASH) score, pain-visual analogue scale (VAS), grip strength, whether they gained a full range of motion (ROM), and complications before and after surgery. Results: Finally, 29 and 28 patients were included in the control and rehabilitation groups, respectively. At final follow-up, the DASH score, grip strength, and ROM were significantly improved in the rehabilitation group compared to that preoperatively. At final follow-up, pain was significantly improved in both groups from that preoperatively. There were no significant differences in the results, including the DASH score, grip strength, ROM and pain-VAS between the control and rehabilitation groups at the final follow-up. Subgroup analysis showed that there is a significant difference in the DASH score of patients doing housework or light work and those with a duration of symptoms >12 months between the control and rehabilitation groups at the final follow-up.

## 1. Introduction

Trigger finger is one of the most common conditions treated by hand surgeons. The prevalence in the general population was reported to be 3% [1]. Several management approaches have been reported for the treatment of trigger finger [2,3]. Corticosteroid injection is a common management strategy in the initial treatment of symptomatic trigger digits. It was reported that 40–80% of patients had a resolution of symptoms following corticosteroid injection [4]. If conservative interventions or corticosteroid injections are unsuccessful, open surgery is generally conducted. Though success rates of open surgical release of the A1 pulley were reported to be around 90%, some patients suffer from postoperative adverse outcomes, including finger stiffness or pain [5,6]. Everding et al. reported that 4.9% of patients had remaining pain, swelling, or stiffness and that 2.5% had residual contracture after open surgery [5].

Some studies revealed the effectiveness of rehabilitation after some kinds of surgeries, including the fixation of extremity fractures and joint replacement [7,8,9]. Rehabilitation is also effective after hand surgeries [10,11,12]. As for trigger finger, it was reported that patients with open surgical release and rehabilitation therapy achieved good results and a low rate of complications [13]. However, this study is not a comparative study but a case series; thus, it includes many biases. Additionally, it is not clear which type of patients should undergo rehabilitation after open surgery. Therefore, we conducted a randomized controlled study with a primary objective to examine the effectiveness of early rehabilitation on hand function and patient-reported outcome (PRO) compared to advice alone. A secondary objective was to identify which patient factors, such as severity of disease, age, duration of symptoms, and type of occupation, influence the effect of rehabilitation.

## 2. Materials and Methods

### 2.1. Design

This study was a prospective, multicenter, randomized, controlled trial (RCT). Approval was obtained from the institutional ethics committee at Okayama University Hospital (1903-002).

The participants were selected from a consecutive group seen at three institutions from April 2019 to March 2021. Inclusion criteria were (1) older than 20 years old, younger than 90 years old; (2) continued subjective symptoms of pain, triggering along the A1 pulley after a few injections into the flexor sheath; and (3) willingness to undergo an operation for trigger finger. We excluded patients who had (1) other fingers with symptoms of trigger finger; (2) an inflammatory or pathologic etiology, including rheumatoid arthritis, of the trigger finger; (3) a finger joint problem by osteoarthritis or joint fracture; or (4) other surgeries during the follow-up period.

### 2.2. Randomization

Participants were assigned to the intervention or control group. An independent investigator (R.N.) made a computer-randomized list for each hospital to conceal the treatment allocation. The randomization list was stratified with a block size of four. Based on the lists, the investigator prepared sequentially numbered and sealed envelopes containing the assigned postoperative schedule. After the participants agreed to participation in this study and underwent surgery for trigger finger, the researcher opened the consecutive envelope to figure out the assigned schedule. Blinding of the participants was precluded because of referral to postoperative rehabilitation.

### 2.3. Treatments

All participants underwent open surgery under local anesthesia by one surgeon. After an incision was made at the level of the A1 pulley, blunt dissection was performed on the A1 pulley. A small dissection scissor was used to make an incision longitudinally and open the A1 pulley. Free movement of the flexor tendon was ensured without triggering. Finally, the skin was closed with interrupted 5-0 nylon sutures. The patients took non-steroidal anti-inflammatory drugs (NSAIDS) on demand after surgery.

### 2.4. Intervention

Participants in the rehabilitation group received a referral for postoperative occupational therapy (OT), such as stretching and an active and passive range of motion exercises within a week after surgery. They also received scar treatment, including friction massage and taping, and strengthening, including the use of grippers, weights, balls with different degrees of firmness, or other aides.

The exact type of exercise was left to the hand therapist’s discretion in accordance with the condition of participants’ hands and their preferences. Additionally, therapists gave appropriate individual advice on activities of daily living and lifestyle for each participant.

Participants in the control group were not referred for rehabilitation after surgery. They received only the orientation and advice for active and passive ROM exercises at distal interphalangeal, proximal interphalangeal, and metacarpophalangeal joints from the primary surgeon during the clinical phase, to be performed by themselves. They were explained that the heel of the hand on the healthy side presses on the back of the hand on the affected side to reach full flexion during passive ROM exercises and that passive extension is also performed using the hand on the healthy side.

### 2.5. Follow-Up

Participants were examined and interviewed before and 1, 3, and 6 months after surgery, and the primary time point was 6 months. The follow-up period was determined by referring to other RCTs of treatments for trigger finger [14,15]. We evaluated subjective symptoms and physical findings, including tenderness at the A1 pulley, maximal grip strength, snapping phenomenon, and whether patients gained full range of motion (ROM) of the treated digit. The Disability of Arm-Shoulder-Hand (DASH) score, pain-visual analogue scale (VAS) of the treated digit, and complications were also examined.

### 2.6. Statistical Analysis

The primary endpoints were the effect of postoperative rehabilitation on hand function, such as DASH and grip power. An estimation of the sample size was performed to determine the number of patients who reached an alpha value of 0.05 and a power of 80%. This sample size was based on the assumed primary outcome (DASH score) differences in population means of 6.0, a within-group standard deviation of 8.0, and an approximately equal number of cases in each group. Finally, the calculated sample size with an anticipated loss to follow-up of 15% was 68 patients.

We performed an intention-to-treat analysis among participants. A participant in the rehabilitation group who converted to the no rehabilitation group was analyzed within the rehabilitation group. The independent investor (R.N.) performed a statistical analysis. Fisher’s exact test and the Student *t*-test were used to compare dichotomous and continuous data, respectively. The group differences were analyzed by one-way ANOVA followed by Bonferroni post hoc testing. Statistical analyses were performed using R for Windows (Ver. 4.0 accessed on 24 April 2020). The two-sided significance level was set at *p* < 0.05.

## 3. Results

### 3.1. Patient Characteristics

**Figure 1:**
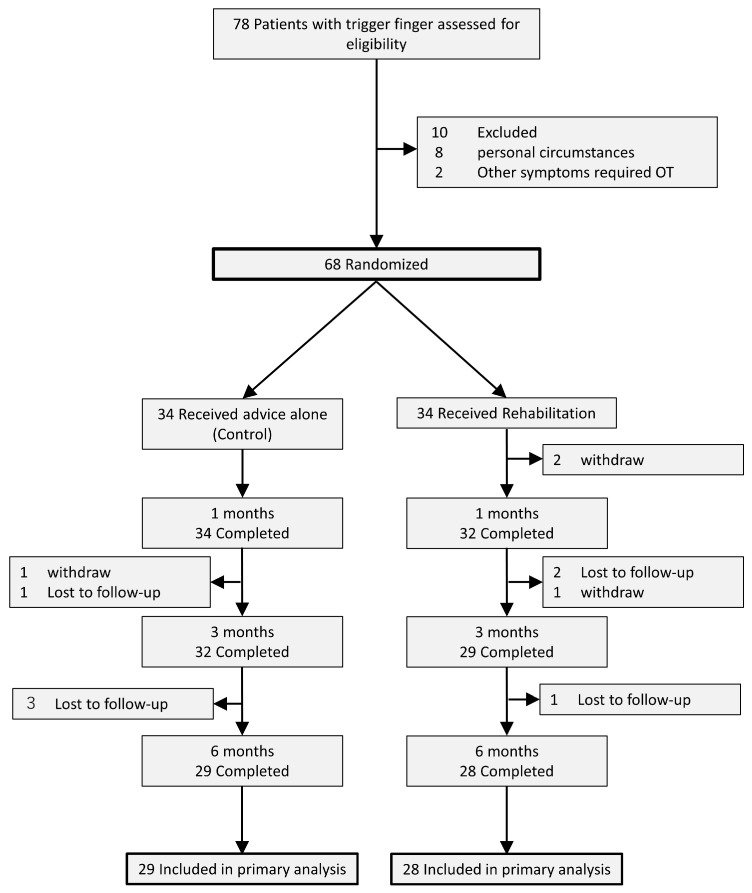
Design and flow of participants through the trial.

Figure 1 shows the flow of the participants through the trial. Initially, we included 68 patients in this study. Thirty-four of them were randomized into the control and rehabilitation groups, respectively. Four patients in the control group and three patients in the rehabilitation group were lost to follow-up by the final examination. One patient in the control group and three patients in the rehabilitation group withdrew by the final examination.

Patient characteristics are shown in Table 1. There were no significant differences between the control and rehabilitation groups in relation to demographic variables, including gender, age, duration of symptoms, preoperative severity of the trigger finger, dominant hand affected, and affected digits. The preoperative severity was classified using the Quinnell grading [16]. The prevalence of diabetes mellitus (DM) and restricted range of motion (ROM) also did not differ significantly between these two groups. DM was well-controlled by oral drugs without insulin treatment in both groups.

### 3.2. Effect of Postoperative Rehabilitation

**Figure 2:**
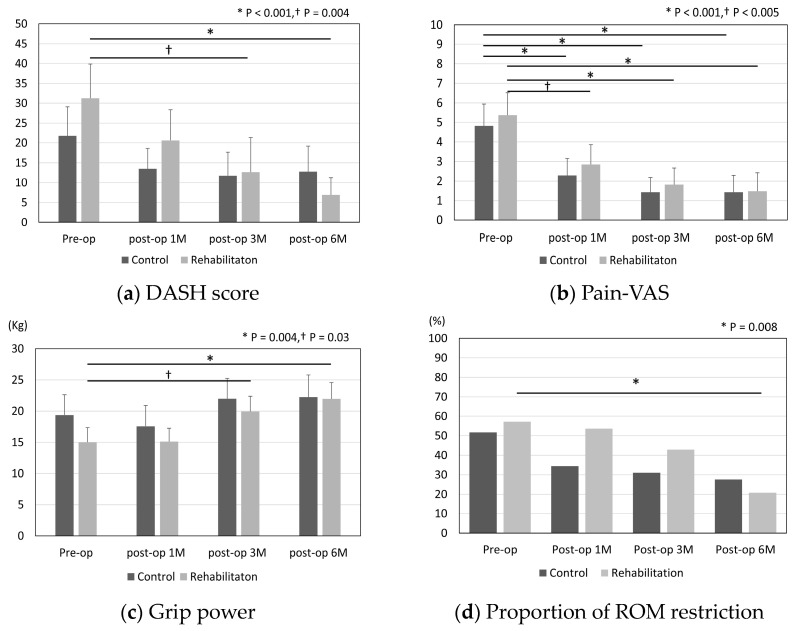
(**a**) DASH score, (**b**) pain-VAS, (**c**) grip power, and (**d**) proportion of ROM restriction. DASH score, The Disability of Arm-Shoulder-Hand score; VAS, visual analogue scale; ROM, range of motion. The bar and error bar in the graphs show the mean and 95% confidence interval. The group differences were analyzed by one-way ANOVA followed by Bonferroni post hoc testing. *p*-values < 0.05 were considered statistically significant.

Figure 2 shows the results for the DASH score, pain-VAS, grip strength, and range of motion in the control and rehabilitation groups. Improvements of the DASH score, grip strength, and ROM were noted significantly in the rehabilitation group at 6 months after surgery (DASH score; *p* < 0.001, grip strength; *p* = 0.004, restriction of ROM; *p* = 0.008).

In both groups, the pain was significantly improved between that occurring preoperatively and 6 months postoperatively. We summarized the differences between pre- and 6-month-postoperative outcomes in Table 2. The grip strength and DASH in the rehabilitation group were improved significantly when compared to those in the control group. However, there were no statistically significant differences in terms of post-operative outcomes, including DASH scores, pain-VAS, grip strength, and ROM restriction, between the control and rehabilitation groups (Supplementary Data, Table S1).

### 3.3. Subanalysis

Rehabilitation made the DASH score in patients performing housework or light work improve significantly 6 months postoperatively (Table 3, 14.2 vs. 6.1, *p* = 0.04). In the patients with a duration of symptoms > 12 months, the postoperative DASH score of the rehabilitation group was also significantly better than that of the control group (Table 4, 19.6 vs. 0.7, *p* = 0.005). There are no statistically significant differences in the subanalysis of other factors, such as age, gender, and DM, between the rehabilitation and control groups.

### 3.4. Complications

One patient in the rehabilitation group had a superficial incision infection that was treated with debridement and an antibacterial drug. There were no other adverse events during this study.

## 4. Discussion

This study showed that the outcomes, including the DASH score, grip power, and restriction of ROM, at 6 months were only significantly improved after surgery in the rehabilitation group. Pain-VAS was significantly improved in both groups. However, there are no significant differences in the outcomes between the control and rehabilitation groups. In the patients performing housework or light work or the patients with a duration of symptoms over 12 months, there were significant differences in the DASH score 6 months after surgery between the two groups.

The effect of postoperative rehabilitation was revealed in some kinds of surgeries [8,11,17]. In hip fracture patients, hospital rehabilitation was significantly related with a lower risk of mortality compared to no rehabilitation [8]. It was also reported that rehabilitation improved mobility for veterans with major lower extremity amputation [17]. As for hand function, rehabilitation after carpal tunnel release improves hand function one month after surgery and accelerates recovery [11]. This study revealed that rehabilitation after surgery significantly improved PRO and hand function, as with other surgeries.

Early motion after upper limb surgery is effective. Many studies demonstrated benefits of early motion, including preventing restriction of ROM, faster healing, decreased disability time, and decreased risk of reflex sympathetic dystrophy [18,19,20]. Additionally, early motion helps edema to decrease, while decreased edema also helps with increased ROM [21]. A meta-analysis that evaluated the effect of rehabilitation following arthroscopic rotator cuff tear repair revealed that early passive motion results in superior ROM recovery [22]. In this study, postoperative rehabilitation was also started within a week, and this early motion by occupational therapists might help improve functional and subjective outcomes.

There were no significant differences in the results, including the DASH score, pain, grip strength, and ROM, between the control and rehabilitation groups in this study. However, both groups could improve these results after surgery. These results imply that simple advice about range of motion exercises may be sufficient for patients after open surgical release.

Some studies have revealed the relationship between long-standing symptoms and postoperative outcomes in relation to various kinds of surgeries [23,24,25]. Inderhaug et al. reported that long-standing symptoms (over 12 months) were identified as one of the predictors of inferior long-term outcome after rotator cuff repair [25]. This study revealed that patients with long-standing symptoms (over 12 months) tended to show worse postoperative DASH scores. Among such patients, the patients with postoperative rehabilitation could show significantly improved DASH scores compared to the patients without rehabilitation. Degenerative thickening of the flexor tendons causes a persistent flexed flexion deformity of the PIP joint [26]. Therefore, postoperative rehabilitation is more important and recommended for patients with long-standing symptoms.

This study also revealed that postoperative rehabilitation for trigger finger was effective for patients performing housework or light work, while there was no significant difference in the postoperative DASH score between those doing heavy manual work with rehabilitation and without it. The postoperative passive and active motion of fingers resulting from doing heavy manual work may act as an adequate range of motion exercises for patients with trigger finger.

DM has an influence on the development of trigger finger [27,28]. It was reported that the incidence of trigger finger in patients with DM was about four times higher than in the general population [29] and that approximately 20% of patients with trigger finger had DM [30]. This study included 15.8% of patients with DM, consistent with previous reports. As for the impact of DM on surgical outcomes, it was revealed that there were no differences in functional and subjective outcomes between diabetic and nondiabetic patients [31]. This study also showed no differences between them. Additionally, rehabilitation had no influence on outcomes for patients with DM in this study. The conditions of all patients with DM were well controlled by oral drugs, which may have had an effect on the outcomes.

This study has some limitations. First, participants could not be blinded due to the nature of the rehabilitation intervention. However, we performed allocation concealment of the participants and minimized the bias in the randomization process. Second, we could not control participants’ uncertainty about the use of their hands after surgery in daily life and pain medication because the patients could take NSAIDS on demand for pain control. We could also not perfectly standardize physiotherapy because the type of exercise was left to the different therapists’ discretion in accordance with the condition of hands. These factors may have influenced the outcomes. Third, this was not a long-term follow-up study. However, it was revealed that there were no statistical differences in clinical outcomes, including VAS and Quick DASH, at 6 months and 12 months after percutaneous or open release for trigger finger [32]. Additionally, several other studies on treatments for trigger finger also set the follow-up period to under 6 months, similarly to this study [14,33,34,35]. In terms of PRO, the DASH score was improved, but not significantly, in the control group. However, the DASH score might be unsuitable to evaluate such a limited hand pathology because the score evaluates the whole upper extremity.

In conclusion, rehabilitation and simple advice after open surgery for trigger finger improved subjective and objective outcomes. Rehabilitation was particularly effective for patients performing housework or light work and for those with long-standing symptoms. The information will help surgeons select patients to refer to OT postoperatively.

## Figures and Tables

**Table 1:** Patient characteristics.

Characteristic	Number			*p*-Value
	Total (n = 57)	Control (n = 29)	Rehabilitation (n = 28)	
Sex				0.25
Men	17	11	6	
Women	40	18	22	
Age	66.5	67.8	65.1	0.41
Duration of symptom (M)	14.3	12.4	16.8	0.46
Quinnell grading				0.08
I	6	2	4	
II	32	20	12	
III	19	7	12	
Dominant hand affected (%)	63.2	62.0	64.2	1.00
Involved digit				0.89
Thumb	17	9	8	
Index finger	3	2	1	
Long finger	17	12	15	
Ring finger	8	5	3	
Small finger	2	1	1	
Diabetes mellitus (%)	15.8	20.7	10.3	0.47
History of trigger finger in other digits (%)	10.5	6.9	14.3	0.42
Restricted range of motion (%)	54.3	51.7	57.1	0.79
Type of occupation				0.39
Housework	17	11	6	
Light work	23	10	13	
Heavy work	17	8	9	

**Table 2:** Differences between pre- and 6-month-postoperative outcomes.

Item	Number		*p*-Value
	Control (n = 29)	Rehabilitation (n = 28)	
Grip strength (kg)	3.8 (0.8–6.9)	8.0 (4.8–11.1)	0.04 *
DASH	6.9 (−2.3–16.0)	23.2 (13.3–33.1)	0.03 *
Pain-VAS	3.7 (2.3–5.1)	3.5 (2.1–4.9)	0.64

* *p* < 0.05.

**Table 3:** Distribution of DASH scores categorized by type of occupation at 6 months after surgery.

Item		DASH (Post-op 6M)	*p*-Value
House or light work	Control (n = 21)	14.2 (5.3–23.0)	0.04 *
	Rehabilitation (n = 19)	6.1 (1.6–10.7)	
Heavy work	Control (n = 8)	9.5 (1.7–17.2)	0.50
	Rehabilitation (n = 9)	8.6 (0–20.8)	

* *p* < 0.05.

**Table 4:** Distribution of DASH scores categorized by duration of symptoms at 6 months after surgery.

Item		DASH (Post-op 6M)	*p*-Value
Duration of symptom < 12 M	Control (n = 12)	5.9 (2.3–9.4)	0.38
	Rehabilitation (n = 15)	9.2 (1.9–16.5)	
Duration of symptom > 12 M	Control (n = 13)	19.6 (7.7–31.5)	0.005 *
	Rehabilitation (n = 6)	0.7 (0–2.5)	

* *p* < 0.05.

## Data Availability

The data presented in this study are available on request from the corresponding author. The data are not publicly available due to patients’ privacy.

## Supplementary Materials

The following supporting information can be downloaded at: https://www.mdpi.com/article/10.3390/jcm12227187/s1, Table S1: Clinical outcomes at 6 months after surgery. The data show the mean and 95% confidential interval.
