# Supplementary material for: The Effectiveness of Rehabilitation after Open Surgical Release for Trigger Finger: A Prospective, Randomized, Controlled Study
Source: J Clin Med. 2023 Nov 20;12(22):7187. doi: 10.3390/jcm12227187 (PMC10671987; doi:10.3390/jcm12227187)
Supplement: Supplementary file 1 [file jcm-12-07187-s001.zip › jcm-2694912-supplementary.pdf]

Supplementary Material

Table S1. Clinical outcomes at 6 months after surgery. The data show the mean and 95% confidential interval.

**Item**	**Number**		**p-Value**
	**Control (n = 29)**	**Rehabilitation (n = 28)**	
Grip strength (kg)	22.2 (18.7–25.8)	22.0 (19.3–24.6)	1.00
DASH	12.8 (6.4–19.2)	6.9 (2.6–11.3)	0.13
Pain-VAS	1.4 (0.6–2.3)	1.5 (0.6–2.4)	0.93
